# Women’s self-care behavior and its relationship with social capital in Yazd, Iran

**DOI:** 10.1186/s12905-021-01469-0

**Published:** 2021-09-13

**Authors:** Hamideh Shiri-Mohammadabad, Seyed Alireza Afshani

**Affiliations:** Department of Cooperative and Social Welfare, Faculty of Social Science, Yazd University, Yazd, Iran

**Keywords:** Social capital, Self-care, Iran, Women

## Abstract

**Background:**

Research on factors affecting self-care is scarce. The social factors, in particular, have not been yet investigated in Iran. Therefore, the present study aimed to investigate the relationship between self-care and social capital among women.

**Methods:**

The participants were 737 women who were living in the marginal, middle and upper areas in the city of Yazd, Iran. Data were collected using a researcher-made self-care questionnaire and Harper’s (Off Natl Stat 11:2019, 2019) Social Capital Scale. The data were analyzed using structural equation modeling by SPSS and Amos v24.

**Results:**

The results showed that the social capital had significant positive effects on the general self-care behavior of the participants (β = 0.56, *p* < 0.001). It also had significant positive effects on the self-care behavior of women living in the marginal (β = 0.58), middle (β = 0.49) and upper (β = 0.62) parts of the city (*p* < 0.001). Besides, the women living in the marginal parts had relatively lower levels of self-care compared to those living in the middle and upper parts of the city. The examination of the fit indices indicated that the model has a good fit (CMIN/DF = 2.087, NFI = 0.921, RMSEA = 0.027, CFI = 0.956, TLI = 0.940, GFI = 0.956, IFI = 0.957).

**Conclusion:**

The findings of this study demonstrated that social capital has significant positive effects on the general self-care behavior of women. Therefore, improving their self-care can be achieved through promoting their social capital.

**Supplementary Information:**

The online version contains supplementary material available at 10.1186/s12905-021-01469-0.

## 1. Background

Self-care is a multidimensional concept that encompasses various aspects of one’s personal life [1, 2]. It has also been conceptualized as a movement [3], a process [4], a set of principles, strategies, or behaviors [2] even abilities [5] with various physical, mental/emotional, spiritual, intellectual, social and recreational dimensions [2, 6–8].

Although there has been an increasing emphasis on self-care in recent years, there is still no general consensus on its definition [7, 9]. Self-care specifically refers to some deliberate, learned and purposeful practices and activities performed by individuals to sustain and promote their health [10–12].

The self-care behavior among women is a highly significant topic in medical sociology and gender studies; however, there is scarce research on it in Iran particularly, in Yazd. With respect to the units of analysis, the dimensions of the self-care and the preventive perspective adopted in the present inquiry, this study can be regarded an initiative investigation into women’s self-care behavior in Iran.

Reports published by the Statistical Center of Iran have provided some examples of Iranian women’s self-care behaviors. A review of such behaviors indicates that women do not appreciate self-care well. For instance, Iranian women above 15 years spend only “12” and “13” minutes per day on “sports” and “cultural and recreational activities”, respectively; while the same people spend a large fraction of their time (on average 5 h and 16 min) on housekeeping activities [13]. Happiness is an indicator of self-care behavior and according to a UN report, Iran is in an unfavorable condition in terms of happiness (score 4.5; rank 117 out of 156) [14].

No matter where one lives, the birth of a boy or a girl usually has significant impact on all the aspects of one’s life, including their health issues; however, the type and the nature of the consequences vary from one society to the other [15]. Gender is held as a socio-cultural criterion, and is considered as one of the basic reasons for inequality in health [16]. Various forms of gender inequality can be seen throughout the world and, in particular, in Iran. Due to the dominance of traditional culture over one’s personal and social life, Iranian women are always the witness of different behaviors from those around them. Girls, for instance, soon give up the childish pursuits, find feminine behaviors and become socially accepted to follow the ideal model of a housewife with several children. Women are often considered less significant than men and occupy lower positions, are less likely to be employed than men, face a variety of barriers and inequalities in the job market. In addition, the women’s biological differences, their different positions in the social structures, their roles and responsibilities, their position in the family, the persisting shadow of traditions on their personal and family lives and their social power are all parts of the problems with which they have to struggle [17]. For instance, the interaction of genetic, hormonal and physiological issues with inadequate social support, financial poverty, the stresses related to childbirth, the mother and child health, and household responsibilities are among the factors that increase the likelihood of unhealthy behaviors in women, the question that arises here now is whether they can take good care of themselves under such circumstances or not.

Researchers have overlooked social factors influencing human behavior when discussing the importance of self-care and the vitality of health as a personal achievement that one acquires through their behaviors [18]. Individuals’ choices depend on the opportunities that life offers them; hence, one cannot gain a proper understanding of self-care behavior without taking the relevant social factors into account. Social capital is among the social determinants of self-care.

The concept of social capital is strongly based on social relationships [19]. It refers to a set of actual or potential resources associated with a cohesive network of more or less institutionalized relationships of mutual acquaintance and recognition [20]. Social capital can affect an individual’s self-care behaviors in different ways. First, it influences one’s health behaviors by enhancing rapid diffusion of health-related information, i.e., individuals with desirable social capital can easily adopt healthy norms and control over their deviant behaviors [21]. Second, social capital reinforces social self-help in societies, and enables them to easily work together to solve collective problems such as health issues [22]. It also affects self-care by facilitating access to self-care services, facilities, knowledge and skills [21, 23]. Finally, a wide range of studies have confirmed the effective role of social capital in improving health and self-care behaviors. For instance, in Marume’s study [24] of the refugees in Zimbabwe, it was found that social capital has a significant role in improving the refugees’ health. In another study on the rural Uttar Pradesh in India by Hasan [25], the results have showed a significant relationship between social capital and self-care. Also, Chong et al. [26] believe that there is a significant positive relationship between social capital and health behaviors among the youth in the suburbs of Malaysia. In line with previous studies, this survey investigated the effectiveness of social capital in improving women’s self-care behavior.

## Methods

### Objectives

This cross-sectional survey assessed the level of self-care in a sample of women in Yazd, Iran and investigated the relationship between women’s social capital and self-care behavior.

### Study population

The study population consisted of all the women living in Yazd. Yazd Municipality has divided the city into different neighborhoods (upper, middle and marginal parts) based on previous studies as well as the neighborhoods’ characteristics such as the quality of the buildings, the socio-economic characteristics of the residents, the residents’ education qualifications, the health and the service indicators [27]. The Sadrabad, Hasanābād and Eskan neighborhoods were considered as the marginal parts; Naiemabad, Azar Yazdi and Razmandegan neighborhoods as the middle parts; and Jomhouri Blvd., Safaeieh and Maskan neighborhoods as the upper parts of Yazd.

The participants were selected from nine different neighborhoods according to the density level in Yazd through a cluster sampling method in proportion to size. To meet this end, at first, the nine neighborhoods in Yazd were considered as nine main clusters. Then, the streets and main squares in each cluster were considered as the block. At last, the participants were randomly selected.

Participants met the inclusion criteria if they were 18–70 years old and were living in the neighborhood for more than 2 years. From each house, only one person was selected. Temporary residence in the neighborhood, unwillingness to participate in the study and the presence of problems such as blindness and deafness were among the exclusion criteria.

The questionnaire was completed at home by the household women at the presence of one of the researchers, who tried to collect a sufficient amount of data with regard to the variables of age, employment status, and their educational level.

In order to estimate the sample size, a pilot study was first conducted on 50 women to determine the variance of self-care. Using Cochran’s sample size formula (error = 5%; 95% CI), the sample size was then determined as 246 for each of the marginal, middle and upper parts in Yazd. To compensate for incomplete questionnaires and loss to follow-up cases, a total of 250 questionnaires were given to eligible participants residing in each part of the city between June and October 2020. A total of 737 legible questionnaires were collected and analyzed.

### Variable measurement

#### Socio-demographic characteristics checklist

A socio-demographic characteristics checklist was used to collect information about the participants’ age (open question), marital status (single, married, or others), educational qualifications (open question) and the number of children (open question).

Social Capital Scale: The social capital was measured through Harper’s Social Capital Scale [28]. This 26-item scale consists of dimensions such as social participation (networks), social networks and social support, reciprocity and trust, civic participation and views of the local part. The items are scored on a five-point Likert scale ranging from 1 to 5. The reliability of the scale is acceptable (Cronbach’s alpha = 0.880).

Self-Care Scale: Various researchers have used different variables to measure self-care. Table 1 shows a selection of them.

**Table 1:** Assessing self-care in some experimental researches

Researcher	Dimensions
Dorociak (2015)	Physical self-care, psychological self-care, spiritual self-care, social self-care and recreational self-care [29]
The Institute for Functional Medicine (2016)	Physical wellbeing, mental/emotional/spiritual wellbeing, professional life/work/career and social life/family/relationships [30]
Clarke (2017)	Physical self-care, emotional self-care, social self-care and professional self-care [2]
Saakvitne and Pearlman (1996)	Physical self-care, psychological self-care, emotional self-care, spiritual self-care and workplace or professional self-care [31]

Considering the operational definitions of the self-care given in research [2, 29–32], in order to measure the self-care in the present study, we recognized that in different questionnaires, only some dimensions of the self-care have been addressed and some items in some questionnaires are not particularly suitable for our society. Therefore, it was decided to combine the dimensions of these questionnaires and prepare a comprehensive questionnaire. In this regard, 12 copies of the questionnaire were provided to seven experts in the field of sociology, three experts in the field of demography and two experts in the field of psychology. They were then asked to comment on the content validity ratio (CVR) of the questionnaire, i.e. the necessity of the items, as well as the content validity index (CVI) of the questionnaire, i.e. "simplicity and fluency", "relevance, or specificity" and "clarity or transparency" of the items based on the objectives of the study. Finally, the items with CVR equal to and greater than 0.75 and CVI equal to and greater than 0.79 remained in the questionnaire. The final questionnaire consisted of 30 items that would examine the physical, psychological/emotional, spiritual, intellectual, social and recreational dimensions of women’s self-care.

Physical self-care (5 items): It is related to the care of the physical self, and incorporates the strategies to optimize physical function and safety [33].

Psychological self-care (5 items): It encompasses the emotional and cognitive strategies to maintain a positive and compassionate view of the self, negotiate the external demands with internal expectations and identify, accept and express a range of emotions [34].

Spiritual self-care (5 items): It includes the search for meaning and purpose in life, the connection to the religious beliefs, values and practices that give meaning to life [35].

Social self-care (5 items): It refers to the strategies to build meaningful and positive relationships and develop a sense of connection, belonging and support [36].

Recreational self-care (5 items): It refers to participation in enjoyable activities that promote relaxation, rejuvenation, or encourage creativity [9].

Intellectual self-care (5 items with reverse scores): It is related to the exploration of ideas and learning in a creative manner [6].

The dimensions of self-care were measured on a five-point scale (1: never, 2: sometimes, 3: often, 4: usually, 5: always). The maximum score was 150 and the minimum score was 30. Higher scores indicated a higher level of self-care. Cronbach's alpha of 0.760 was obtained on this scale (Additional file 1).

### Statistical analysis

The socio-demographic variables were analyzed using descriptive statistics (including percentage, frequency, mean and standard deviation). A one-sample t-test was used to compare the differences regarding the participants’ self-care levels. Using a structural model, the authors assessed the relationships between the social capital and the self-care behavior among the women residing in the marginal, middle and upper parts of the city. They also compared the intensity of the relationships between the research variables in different parts. The data were analyzed in SPSS Amos v24 using structural equation modeling (SEM).

## Results

### Respondents’ characteristics

Of the total of 737 participants in the present study, 558 women (75.7%) were married and, by compartmentalizing the parts, married women comprised the majority of women living in the marginal, middle, and upper parts.

The results of the present study revealed that the mean and standard deviation of education qualifications were 12.83 ± 3.71. The results also indicated that the women living in the marginal parts had the lowest mean scores (10.83 ± 3.75). In terms of the number of children, the mean and standard deviation were 1.85 ± 1.61. The findings also indicated that the women living in the marginal parts had the highest mean scores (2.14 ± 1.75) compared to those living in the middle and upper parts of the city.

In addition, the results of the one-way ANOVA showed that there is a significant difference between the women living in the upper, middle and marginal parts of Yazd in terms of the educational qualifications and the number of children (*p* < 0.01); however, no age difference was observed between women living in the upper, middle and marginal parts of Yazd (*p* ˃ 0.05). The results of the chi-square test showed that there is a significant difference between the women’s marital status in different parts, and the percentage of married people in the marginal parts was significantly higher than women living in the upper parts (*p* < 0.001). The demographic characteristics of the respondents are presented in Table 2.

**Table 2:** respondents’ characteristics

Variable	Parts	Mean	SD	*p*-value
Age	Marginal	35.83	10.85	0.373
	Middle	35.74	11.45	
	Upper	34.53	11.67	
	Full sample	35.37	11.33	
Education qualifications	Marginal	10.83	3.75	0.001
	Middle	13.62	3.43	
	Upper	13.96	3.12	
	Full sample	12.83	3.71	

Marital status	Parts	n	%	*p*-value
Married	Marginal	204	83.3	0.001
	Middle	189	76.8	
	Upper	165	67.3	
	Full sample	558	75.7	
Single	Marginal	33	13.5	
	Middle	55	22.4	
	Upper	73	29.8	
	Full sample	161	21.9	
Others	Marginal	8	3.3	
	Middle	2	0.8	
	Upper	7	2.9	
	Full sample	17	2.3	

	Parts	Mean	SD	*p*-value
Number of children	Marginal	2.14	1.75	0.004
	Middle	1.71	1.50	
	Upper	1.71	1.56	
	Full sample	1.85	1.61	

*SD* standard deviation; variable ranges: age (18–70); marital status (1–3); education qualifications (0–20); number of children (0–11); marginal part (n = 246); middle part (n = 246); upper part (n = 245); full sample (n = 737)

Based on the results of the one-sample t-test, the minimum and maximum self-care scores were 30 and 150, respectively; thus, the mean score of women’s self-care in the marginal, middle and upper parts of Yazd was higher than 90 (theoretical mean). Therefore, the overall level of the participants’ self-care was relatively above the average (*p* < 0.001).

In addition, the one-sample t-test was used in order to answer the question "How is the social capital of the women living in the marginal, middle and upper parts of Yazd?". To assess the social capital among the respondents, 24 items were included in the questionnaire. Given that each item had five options, the minimum and the maximum scores were 24 and 120, respectively. The score 72 was chosen as the theoretical mean. A score of higher than 72 indicates that the respondents’ level of social capital is higher than average and a score of lower than 72 indicates that the respondents’ level of social capital is lower than average. The result of the one-sample t-test showed that the mean score of the social capital of the women living in the marginal, middle and upper parts was higher than 72 (the theoretical mean). Therefore, in total, the respondents’ social capital was above the average (*p* < 0.001) (Table 3).

**Table 3:** The output of one-sample t-test on the differences of self-care and social capital of women in the marginal, middle and upper parts of Yazd

Variable	Part	Mean (SD)	Mean difference	t	*p*-value
Self-care	Marginal	94.66 ± 11.49	4.66	6.358	0.001
	Middle	97.21 ± 12.37	7.21	9.142	0.001
	Upper	100.20 ± 14.15	10.20	11.291	0.001
Social capital	Marginal	83.15 ± 15.18	11.15	11.52	0.001
	Middle	91.67 ± 18.53	19.67	16.65	0.001
	Upper	87.93 ± 14.77	15.93	16.88	0.001

An independent-sample t-test was also used to compare self-care among employed and unemployed women in the three upper, middle and marginal parts in the city. The results showed no significant difference (*p* ˃ 0.05) (Table 4).

**Table 4:** The output of t-test on the differences of self-care of women in the marginal, middle and upper parts of Yazd

Variable	Part	Groups	Mean (SD)	Mean difference	t	*p*-value
Self-care	Marginal	Employee	96.17 ± 9.00	1.48	1.003	0.317
		Unemployed	94.69 ± 10.00			
	Middle	Employee	96.55 ± 11.68	− 0.67	− 0.438	0.662
		Unemployed	97.23 ± 11.29			
	Upper	Employee	101.20 ± 12.29	1.49	0.687	0.493
		Unemployed	100.17 ± 11.09			

### The structural model of the self-care

**Fig. 1:**
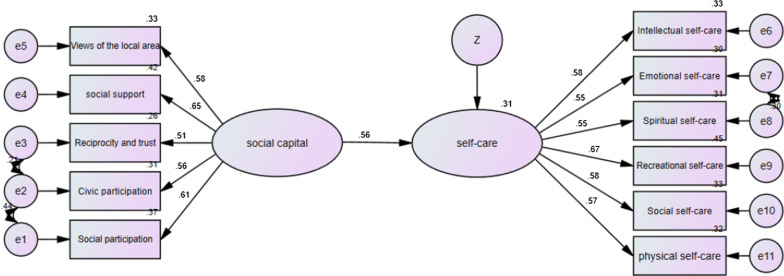
The model of the relationship between social capital and self-care in the full sample

**Fig. 2:**
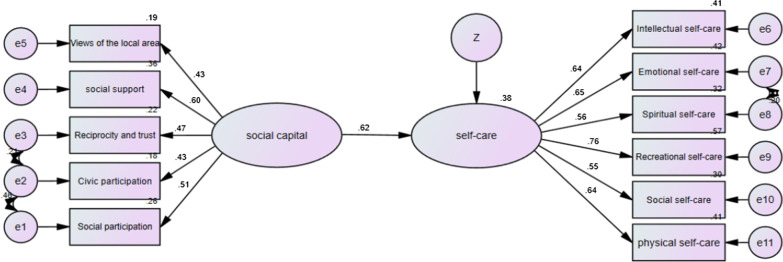
The model of the relationship between social capital and self-care in the upper parts of Yazd

**Fig. 3:**
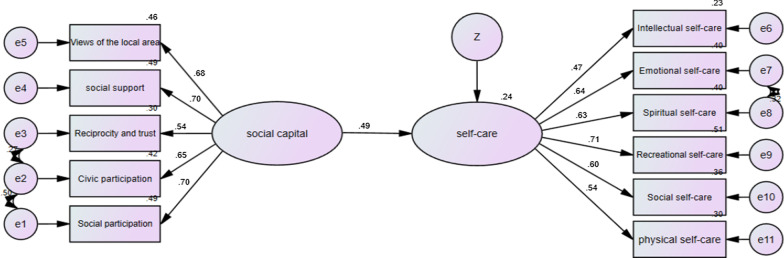
The model of the relationship between social capital and self-care in the middle parts of Yazd

**Fig. 4:**
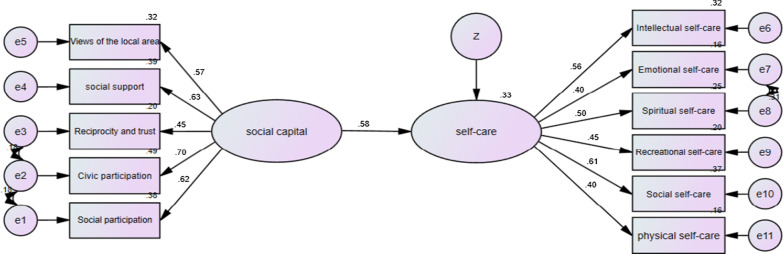
The model of the relationship between social capital and self-care in the marginal parts of Yazd

Using the structural model, the relationship between the social capital and the self-care was examined separately in different parts of Yazd, the outputs were interpreted, and the coefficients of these three groups were compared (Figs. 1, 2, 3, 4).

As shown in Table 5, the social capital had significant positive effects on the general self-care behavior of the participants (β = 0.56, *p* < 0.001). It also had significant positive effects on the self-care behavior of women living in the marginal (β = 0.58), middle (β = 0.49) and upper (β = 0.62) parts of the city (*p* < 0.001).

**Table 5:** Significance of causal pathways of Structural equation model of social capital and self-care based on parts

Part	Path	Weights	C.R	*p*	Result
Marginal	Social capital → Self-care	0.575	4.667	0.001	Significant
Middle	Social capital → Self-care	0.487	4.518	0.001	Significant
Upper	Social capital → Self-care	0.617	4.246	0.001	Significant
Total	Social capital → Self-care	0.559	8.284	0.001	Significant

***p < 0.001 (two-tailed); *C.R.* critical ratio

In addition, we used the model fit indexes to estimate the model fit, and the models fit the data adequately (CMIN/DF = 2.087, NFI = 0.921, RMSEA = 0.027, CFI = 0.956, TLI = 0.940, GFI = 0.956, IFI = 0.957, see Table 6).

**Table 6:** The fit indices of the structural equation model of social capital and self-care

Indexes	CMIN	DF	CMIN/DF	GFI	NFI	IFI	TLI	CFI	RMSEA
Values	333.995	160	2.087	0.961	0.921	0.957	0.940	0.956	0.027

## Discussion

Social capital is an important determining factor in population health [18, 37, 38]. It can influence the self-care behavior through several possible causal mechanisms including the influence of social cohesion on health-related behaviors, differential procurement of social services, access to resources such as emotional and material support, health information generated by social networks and variations in individuals’ access to life opportunities and material resources (e.g., health care, education) [39, 40]. Besides, there are factors like socioeconomic factors (e.g., inequality), social network structures (e.g., homogeneity, density) and characteristics of network ties (e.g., frequency of contact, strong/weak ties, reciprocity) which can affect the type of the social capital that is being formed and the individuals’ health behaviors [38]. Considering the importance of this issue, the present study aimed to investigate the explanatory role of the social capital on the self-care behaviors of women living in the marginal, middle and upper parts of Yazd.

The results of our study showed that the level of self-care among the women living in the marginal, middle and upper parts of Yazd was above the average and the women living in the upper parts had a higher level of self-care compared to the women living in the marginal and middle parts (*p* < 0.001).

The self-care behaviors in different strata and groups are the result of a set of choices that a person chooses based on their social status and life opportunities. In fact, from Weber's point of view, the self-care behavior is the result of a dialectical relationship between life opportunities and lifestyle. Life opportunities are considered as a form of structure that basically reflects the status of one’s social class, and weakens and limits their choices. In other words, those strata of the society who possess a good life opportunity will be able to participate in physical activities and have more opportunities for relaxation and eat healthier food. However, those strata of the society (for example, the residents of the marginal parts) who do not possess a good position in the social classification will have unhealthier performances due to the constraints that poor life opportunities create for them [41]. According to the results of the present study and the relevant studies [41, 42], it seems that the individuals’ position in the social situations has an important role in the possibility or impossibility of the healthy performances in their behaviors. Also, in the present study, the social capital of the women living in the marginal, middle and upper parts of Yazd was above the average and the women living in the middle parts of the city had the highest and the women living in the marginal parts had the lowest level of social capital (*p* < 0.001).

In Bourdieu's view, the social capital is inequitably distributed by social classes and inextricably linked to the economic and other resources in a reinforcing cycle, such that social capital can further contribute to inequalities [43].

In fact, it is the social class that affects the degree to which individuals have access to social capital. For instance, the individuals in the lower classes of the society, who are forced to live in the marginal parts due to the lack of adequate economic opportunities, often have lower education and do not participate in many social and cultural affairs. Also, under the influence of the experiences of widespread inequalities in the society, a kind of pessimism towards different matters has been formed in them, and, therefore, they have low social trust. Thus, their social capital is below the other people in the society who have a better social class and opportunity to live. In contrast, the people in the middle and upper parts will be able to have constructive social relationships with others due to their higher education qualifications, better jobs and higher incomes, and because of a spirit of participation, they will have the opportunity to spend their leisure time with friends, extensive civic engagement, participation in the social and career plans, and having fun with the group. Each of which is an example of the social capital.

Our findings are in line with previous studies which also confirm a significant effect for the social capital on self-care (β = 0.56, *p* < 0.001). Chong et al. [26] showed that the more social capital young people who live in the marginal areas of Malaysia enjoy, the more likely they are to develop the health behaviors that individuals should display. In addition, Tofani et al. [44] reported that improving the level of social capital among Brazilian women improved their health behaviors. Similarly, Marume et al. [24] showed that social capital had a significant role in improving the health of Zimbabwean asylum seekers.

Social capital consists of different dimensions one of which is social network that plays a significant role in people’s health. The number of support networks and the quality of interpersonal communication in these networks can affect different aspects of people's lifestyles. For example, people who join valuable social networks are less likely to engage in abnormal social behaviors such as excessive drinking [45]. It plays a role both in maintaining the physical self-care of individuals and in promoting their social and psychological self-care by receiving emotional and social support from network members, changing people's attitudes and behaviors towards different issues, as the result of constructive interactions with others. Furthermore, social capital can influence people's self-care behavior through local norms. For example, individuals living in a specific community will be influenced by the behavioral norms of surrounding people. That is to say, if neighbours regularly participate in physical activity (e.g., jogging, cycling), it may increase the likelihood of their participation in physical exercise [46].

Furthermore, the scientific evidence shows that in order to achieve the desired level of self-care among women, a set of basic conditions (e.g. environmental conditions, individuals’ social capital) are involved leading to an outcome in health through certain causal mechanisms [47]. From Bourdieu's perspective, the self-care behaviors among the women are the product of their acquired habits in the social situations they occupy. In fact, their self-care behaviors are determined by the resources (for instance, the social capital and the environmental conditions) available to them. These are among the basic conditions for the realization of self-care behaviors.

When the field (the place where women live) is mentioned, both the class position and the field’s environmental and social characteristics are emphasized, which are a kind of resources, because by being in a certain environmental situation, a person is exposed to certain social and environmental factors that shape their behaviors [48]. Social capital, as one of the resources of the field, plays an important role in shaping the behavioral tendencies of self-care among women. In Bourdieu's view, women choose how to act for their self-care, but they do not have the complete freedom to do so. Their choices are limited by the strong influence of the resources (such as the social capital and the environmental conditions) available in the field [42]. The resources available in the field point to the possibility of the self-care strategies in women by shaping the mental habits (tastes and desires) of women living in the upper, middle and marginal parts of the city.

Not only do the residents of the upper parts of the city have stronger economic capital, but they also have opportunities especially social capital, separating themselves from others. The higher level of social capital for women living in the upper parts of the city provides them with a good background for constructive social relationships and access to information and emotional support. Thus, the women will be able to improve their health behaviors depending on the type of information and emotional support, and even the nature of the social network they belong to. Also, the environmental conditions have a special social meaning and can play an important role in shaping the type and the nature of women's social capital by providing the suitable opportunities such as the desired recreational-cultural atmosphere, and affect their women’s self-care performance. In addition, a safe neighborhood can be a good opportunity for women to walk in and have physical activities. In this way, women can gain emotional support and constructive social interaction in order to perform better regarding their self-care [49, 50].

It is to be noted that there are confounding factors that can influence the effect of social capital on self-care behavior. For example, in the present research, the socioeconomic conditions influenced the individual social capital and thus are considered confounding factors [44]. Therefore, the upper, middle and marginal areas of the city were divided according to the economic and social characteristics to neutralize the effect of confounding factors.

Our findings showed no significant difference between the mean self-care behavior for the employed and non-employed women living in the upper, middle and suburbs (*p* ˃ 0.05). In a similar vein, Atashpeikar et al. [51] also showed that there is no significant difference between the self-care of the employed and non-employed. On the contrary, Tabrizi et al. [52] showed that there is a significant difference between individuals' self-care ability and their employment status. This finding can be interpreted to some extent according to the cultural conditions of Iranian society. There seems to be a counterbalance to the positive and negative effects that employment or non-employment has on self-care behavior [53]. For example, job opportunities greatly affect women's self-care behavior by expanding their social interactions, social support, financial independence, and improvement of their knowledge and skills [29]. Of course, these opportunities are created when the working environment and conditions for women are suitable because the mismatch (incompatibility) between the person and the work environment and even the experience of inequality in the workplace will lead to stress and less life satisfaction in women. On the other hand, housewives can spend more time having fun with their family members because they are solely in charge of the household, do not experience job stress, do not have to endure work-family conflict, and have more time to relax. They can therefore have better self-care. Thus, the employment or non-employment of women creates a situation that will have a positive effect on the self-care behavior of both groups.

## Research limitations

Because the data were based on self-reported answers by the participants, the responses to some items might have been influenced by society values. Another limitation was the scarcity of academic literature on the relationship between social capital and self-care since the theorists do not explicitly address the relationship between social capital and self-care, but argue that social capital plays a decisive role in the individuals' health behaviors.

## Conclusions

This study was an attempt to investigate the effects of social capital on the self-care practiced by women in the upper, middle and marginal areas in the city of Yazd, Iran. The two latent variables of social capital and self-care were the main components of the structural equation model in the present study. The findings indicate that social capital has a positive and significant effect on the self-care behavior of women living in the upper, middle and marginal areas of the city. The greater the amount and extent of social capital are for women, the better they will perform in self-care. Therefore, it is worthwhile to pay attention to this variable in the preparation and development of health promotion programs.

## Supplementary Information

**Additional file 1.** Questionnaire of self-care.

## Data Availability

The datasets used and/or analyzed during the current study are available from the corresponding author on reasonable request.
