# Supplementary material for: Women’s self-care behavior and its relationship with social capital in Yazd, Iran
Source: BMC Womens Health. 2021 Sep 13;21:331. doi: 10.1186/s12905-021-01469-0 (PMC8438965; doi:10.1186/s12905-021-01469-0)
Supplement: Supplementary file 1 — Additional file 1. Questionnaire of self-care. [file 12905_2021_1469_MOESM1_ESM.docx]

| **items** | **never** | **sometimes** | **often** | **usually** | **always** |
| --- | --- | --- | --- | --- | --- |
| 1. Reading a book about something that you wouldn’t normally read about. | 1 | 2 | 3 | 4 | 5 |
| 1. Watching a documentary. | 1 | 2 | 3 | 4 | 5 |
| 1. Doing crosswords or Sudoku puzzles. | 1 | 2 | 3 | 4 | 5 |
| 1. Signing up for a class. | 1 | 2 | 3 | 4 | 5 |
| 1. Learning a new language. | 1 | 2 | 3 | 4 | 5 |
| 1. Making a conscious effort to appreciate positive things in the life. | 1 | 2 | 3 | 4 | 5 |
| 1. Dealing with negative emotions by changing the way I think about the situation. | 1 | 2 | 3 | 4 | 5 |
| 1. Cherishing your optimism and hope. | 1 | 2 | 3 | 4 | 5 |
| 1. Using the sense of humor to keep things in perspective. | 1 | 2 | 3 | 4 | 5 |
| 1. Sharing the feelings with others during stressful times in your life. | 1 | 2 | 3 | 4 | 5 |
| 1. Spending time in nature. | 1 | 2 | 3 | 4 | 5 |
| 1. Feeling good about forgiving others. | 1 | 2 | 3 | 4 | 5 |
| 1. Feeling satisfied with helping and giving gifts. | 1 | 2 | 3 | 4 | 5 |
| 1. Having hope that things will get better. | 1 | 2 | 3 | 4 | 5 |
| 1. Enjoying watching the sunrise and sunset. | 1 | 2 | 3 | 4 | 5 |
| 1. Taking time for recreational or leisure activities. | 1 | 2 | 3 | 4 | 5 |
| 1. Setting time to relax and unwind. | 1 | 2 | 3 | 4 | 5 |
| 1. Striving for balance among work, family, relationships, play, and rest. | 1 | 2 | 3 | 4 | 5 |
| 1. Taking time to relax throughout the day. | 1 | 2 | 3 | 4 | 5 |
| 1. Engaging in a hobby, or a recreational/social activity that I enjoy. | 1 | 2 | 3 | 4 | 5 |
| 1. Having the ability to comfortably say no. | 1 | 2 | 3 | 4 | 5 |
| 1. Sharing a fear, hope, or secret with someone I trust. | 1 | 2 | 3 | 4 | 5 |
| 1. Spending enough time interacting with people who make you happy. | 1 | 2 | 3 | 4 | 5 |
| 1. Staying in contact with faraway friends. | 1 | 2 | 3 | 4 | 5 |
| 1. Having someone you can rely on if you need help or guidance. | 1 | 2 | 3 | 4 | 5 |
| 1. Being sensitive about your weight. | 1 | 2 | 3 | 4 | 5 |
| 1. Drinking enough water. | 1 | 2 | 3 | 4 | 5 |
| 1. Dancing, swimming, walking, running, playing sports, singing, or doing a physical activity. | 1 | 2 | 3 | 4 | 5 |
| 1. Sleeping at least 7 hours per night. | 1 | 2 | 3 | 4 | 5 |
| 1. Consuming a healthy balance of fruits, vegetables, grain, fats, and protein. | 1 | 2 | 3 | 4 | 5 |
